# Oncofertility counselling in premenopausal women with HER2-positive breast cancer

**DOI:** 10.18632/oncotarget.26565

**Published:** 2019-01-29

**Authors:** Matteo Lambertini, Isabelle Demeestere, Giulia Viglietti, Evandro de Azambuja

**Affiliations:** ^1^ Department of Medical Oncology, Institut Jules Bordet and Université Libre de Bruxelles, Brussels, Belgium; ^2^ Breast Cancer Translational Research Laboratory, Institut Jules Bordet and Université Libre de Bruxelles, Brussels, Belgium; ^3^ Fertility Clinic, CUB-Hôpital Erasme and Research Laboratory on Human Reproduction, Université Libre de Bruxelles, Brussels, Belgium

**Keywords:** breast cancer, fertility, HER2, trastuzumab, lapatinib

## Abstract

A complete oncofertility counselling should be offered to all premenopausal patients before the administration of anticancer treatments. This important discussion is a crucial step for allowing them to take fully informed decisions about the proposed therapy and its potential long-term consequences as well as on their need and interest of accessing the available strategies for ovarian function and/or fertility preservation. In premenopausal women with HER2-positive early breast cancer, limited evidence exists to counsel them about the potential added gonadotoxicity of targeted agents beyond the damage already caused by chemotherapy. In addition, the prognostic role of treatment-induced amenorrhea in this setting was unknown. In our exploratory analysis within the ALTTO (BIG 2-06) trial, we have recently described the rates of treatment-induced amenorrhea after chemotherapy plus trastuzumab and/or lapatinib and the prognostic value of developing this side effect according to the hormone receptor status of their tumours. We observed similar rates of treatment-induced amenorrhea in the four anti-HER2 treatment arms. The lack of an increased rate of treatment-induced amenorrhea in the dual anti-HER2 blockade arm suggests the possible gonadal safety of these agents. In addition, women with HER2-positive/hormone receptor-positive tumours showed significantly better survival outcomes if they developed treatment-induced amenorrhea, while no difference was observed for those with HER2-positive/hormone receptor-negative disease. Future research efforts are needed to address the gonadotoxicity of the new available targeted agents as well as to elucidate which is the best adjuvant endocrine therapy in premenopausal women with HER2-positive/hormone receptor-positive disease.

During their initial work-up, all premenopausal women candidates to receive chemotherapy for a newly diagnosed breast cancer should receive a complete oncofertility counselling as part of the current standard of care in order to be informed about the possible gonadotoxic risk associated with the administration of the proposed anticancer treatments [1]. This important discussion is a crucial step for allowing them to take fully informed decisions about the proposed therapy and its potential long-term consequences as well as on their need and interest of accessing the available strategies for ovarian function and/or fertility preservation.

Menstrual function is not a perfect indicator of the actual treatment-induced gonadal damage that can potentially lead to premature ovarian insufficiency, infertility and early menopause; nevertheless, amenorrhea usually represents the first signal of chemotherapy toxic effects on the ovaries [2]. Experiencing treatment-induced amenorrhea has been shown to have a positive prognostic impact with improved outcomes in patients with hormone receptor-positive breast cancer by causing a subsequent state of ovarian function suppression [3]. However, this can also negatively impact on women’s quality of life considering its associated unpleasant menopause-related symptoms and signs that also include possible fertility loss [2]. In premenopausal women with HER2-positive early breast cancer candidates to undergo chemotherapy plus 1-year of trastuzumab-based anti-HER2 treatment as per standard of care [1], limited evidence exists to counsel them about the potential added risk of gonadotoxicity with the administration of targeted agents beyond the damage already caused by systemic cytotoxic therapy. In addition, the prognostic role of treatment-induced amenorrhea in this setting was unknown.

With the final goal to provide useful information for improving the oncofertility counselling of premenopausal women with HER2-positive early breast cancer, we have recently performed an exploratory analysis within the Adjuvant Lapatinib and/or Trastuzumab Treatment Optimization (ALTTO; BIG 2-06) trial aiming to address these unmet medical issues [4]. Specifically, by including 2,862 patients who were premenopausal at randomization, we described the rates of treatment-induced amenorrhea after approximately 9 months following the completion of anthracycline- and/or taxane-based chemotherapy in the four anti-HER2 treatment arms of the trial (trastuzumab alone, lapatinib alone, their sequence or their combination). In addition, we assessed the prognostic value of treatment-induced amenorrhea in patients with HER2-positive early breast cancer according to the hormone receptor status of their tumours [4].

The rates of treatment-induced amenorrhea were similar in the four anti-HER2 treatment arms, being 72.6% with trastuzumab alone, 74.0% with lapatinib alone, 72.1% with trastuzumab followed by lapatinib and 74.8% with dual anti-HER2 blockade of trastuzumab plus lapatinib (Figure 1) [4]. Notably, the ALTTO trial did not have a treatment arm without anti-HER2 therapies; therefore, the answer on the potential added gonadotoxic effect of trastuzumab and/or lapatinib beyond chemotherapy could not be addressed in our study. However, as compared to the trastuzumab alone arm, no increased rates of treatment-induced amenorrhea was observed in the dual anti-HER2 blockade arm (odds ratio, 1.19; 95% confidence intervals [CI], 0.94-1.51; *p* = 0.14) suggesting the possible gonadal safety of these agents, which is in line with previously small studies [5,6]. Therefore, the first main message from our analysis is that, during the oncofertility counselling of premenopausal women with HER2-positive early breast cancer, the discussion around the risk of gonadotoxicity with the proposed treatment as well the need for having access to the available strategies for ovarian function and/or fertility preservation should be driven mostly by the type of chemotherapy regimen proposed and by the age of the patient (i.e., the two most important determinants of this risk). For patients who undergo temporary ovarian suppression with a gonadotropin-releasing hormone agonist during chemotherapy as a mean to reduce the risk of treatment-induced gonadal damage [7], our data (indirectly) do not support the prolongation of such treatment beyond systemic cytotoxic therapy and until the completion of 1-year of anti-HER2 therapy. Future research efforts in the field are warranted to better elucidate the real impact of targeted agents including pertuzumab (recently approved for women with HER2-positive early breast cancer) on women’s ovarian reserve (e.g. by evaluating the dynamic of anti-mullerian hormone levels before and after treatment [8]). This is crucial information to be retrieved also considering the recent reassuring data on the safety and feasibility of conceiving after prior history of breast cancer including among premenopausal women with HER2-positive disease previously exposed to chemotherapy and anti-HER2 treatments [9].

**Figure 1:**
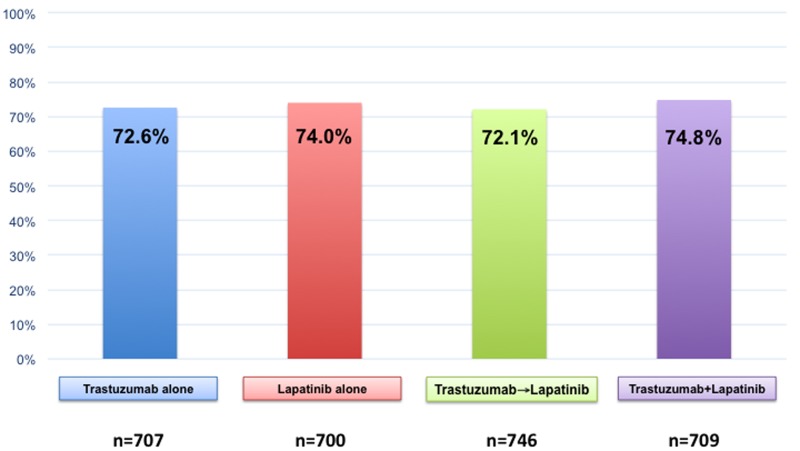
Rates of treatment-induced amenorrhea after chemotherapy plus trastuzumab and/or lapatinib among premenopausal women enrolled in the ALTTO (BIG 2-06) trial.

Regarding the prognostic impact of treatment-induced amenorrhea in premenopausal women with HER2-positive early breast cancer, our study showed a significant interaction of its effect according to hormone receptor status [4]. Specifically, neither difference in disease-free survival (adjusted hazard ratio [HR], 0.92; 95% CI, 0.70 – 1.20) nor in overall survival (adjusted HR, 1.03; 95% CI, 0.68 – 1.56) were observed between patients with HER2-positive/hormone receptor-negative tumours with or without treatment-induced amenorrhea. On the contrary, among women with HER2-positive/hormone receptor-positive tumours, disease-free survival (adjusted HR, 0.58; 95% CI, 0.45 – 0.76) and overall survival (adjusted HR, 0.63; 95% CI, 0.40 – 0.99) were significantly better for patients who developed treatment-induced amenorrhea. Notably, the majority (67.4%) of premenopausal women with HER2-positive/hormone receptor-positive breast cancer included in the ALTTO trial received tamoxifen alone as adjuvant endocrine therapy. The recently updated SOFT trial results showed that the addition of ovarian function suppression to tamoxifen is of particular benefit for the subgroup of women with HER2-positive/hormone receptor-positive disease (p_interaction_ = 0.04) [10]. Our results indirectly support the important role of ovarian function suppression as part of adjuvant endocrine therapy in premenopausal women with HER2-positive early breast cancer. In this setting, the best oral endocrine partner to associate with ovarian function suppression remains unclear [11–13]. As shown in the SOFT and TEXT trial update, ovarian function suppression plus tamoxifen demonstrated a better performance than its combination with an aromatase inhibitor in the subgroup of premenopausal women with HER2-positive/hormone receptor-positive disease (i.e., an opposite trend as compared to the group of women with HER2-negative/hormone receptor-positive breast cancer) [10]. Similar findings have been recently shown also in the HOBOE trial [14]. However, no strong conclusion can be made considering the small number of patients with HER2-positive disease that were included in these trials. Future research efforts are awaited to answer the important question on which is the best oral endocrine partner to combine with ovarian function suppression as adjuvant treatment for premenopausal women with HER2-positive/hormone receptor-positive disease.

## References

[1] Paluch-Shimon S, Pagani O, Partridge AH, Abulkhair O, Cardoso MJ, Dent RA, Gelmon K, Gentilini O, Harbeck N, Margulies A, Meirow D, Pruneri G, Senkus E (2017). ESO-ESMO 3rd international consensus guidelines for breast cancer in young women (BCY3). Breast.

[2] Lambertini M, Goldrat O, Clatot F, Demeestere I, Awada A (2017). Controversies about fertility and pregnancy issues in young breast cancer patients: current state of the art. Curr Opin Oncol.

[3] Zhao J, Liu J, Chen K, Li S, Wang Y, Yang Y, Deng H, Jia W, Rao N, Liu Q, Su F (2014). What lies behind chemotherapy-induced amenorrhea for breast cancer patients: a meta-analysis. Breast Cancer Res Treat.

[4] Lambertini M, Campbell C, Bines J, Korde LA, Izquierdo M, Fumagalli D, Del Mastro L, Ignatiadis M, Pritchard K, Wolff AC, Jackisch C, Lang I, Untch M (2019). Adjuvant Anti-HER2 Therapy, Treatment-Related Amenorrhea, and Survival in Premenopausal HER2-Positive Early Breast Cancer Patients. J Natl Cancer Inst.

[5] Abusief ME, Missmer SA, Ginsburg ES, Weeks JC, Partridge AH (2010). The effects of paclitaxel, dose density, and trastuzumab on treatment-related amenorrhea in premenopausal women with breast cancer. Cancer.

[6] Ruddy KJ, Guo H, Barry W, Dang CT, Yardley DA, Moy B, Marcom PK, Albain KS, Rugo HS, Ellis MJ, Shapira I, Wolff AC, Carey LA (2015). Chemotherapy-related amenorrhea after adjuvant paclitaxel-trastuzumab (APT trial). Breast Cancer Res Treat.

[7] Lambertini M, Moore HC, Leonard RC, Loibl S, Munster P, Bruzzone M, Boni L, Unger JM, Anderson RA, Mehta K, Minton S, Poggio F, Albain KS (2018). Gonadotropin-Releasing Hormone Agonists During Chemotherapy for Preservation of Ovarian Function and Fertility in Premenopausal Patients With Early Breast Cancer: A Systematic Review and Meta-Analysis of Individual Patient -Level Data. J Clin Oncol.

[8] Fréour T, Barrière P, Masson D (2017). Anti-müllerian hormone levels and evolution in women of reproductive age with breast cancer treated with chemotherapy. Eur J Cancer.

[9] Lambertini M, Martel S, Campbell C, Guillaume S, Hilbers FS, Schuehly U, Korde L, Azim HA, Di Cosimo S, Tenglin RC, Huober J, Baselga J, Moreno-Aspitia A (2019). Pregnancies during and after trastuzumab and/or lapatinib in patients with human epidermal growth factor receptor 2-positive early breast cancer: analysis from the NeoALTTO (BIG 1-06) and ALTTO (BIG 2-06) trials. Cancer.

[10] Francis PA, Pagani O, Fleming GF, Walley BA, Colleoni M, Láng I, Gómez HL, Tondini C, Ciruelos E, Burstein HJ, Bonnefoi HR, Bellet M, Martino S, SOF T and TEXT Investigators and the International Breast Cancer Study Group (2018). Tailoring Adjuvant Endocrine Therapy for Premenopausal Breast Cancer. N Engl J Med.

[11] Lambertini M, Viglietti G, de Azambuja E (2018). Controversies in oncology: which adjuvant endocrine therapy is to be given to premenopausal patients with hormone receptor-positive breast cancer?. ESMO Open.

[12] Lambertini M, Viglietti G, de Azambuja E (2019). Impact of ovarian function suppression in premenopausal women with estrogen receptor-positive early breast cancer. Curr Opin Oncol.

[13] Poggio F, Lambertini M, Bighin C, Conte B, Blondeaux E, D’Alonzo A, Dellepiane C, Boccardo F, Del Mastro L (2018). Management of young women with early breast cancer. ESMO Open.

[14] Perrone F, De Laurentiis M, De Pl acido S, Orditura M, Cinieri S, Riccardi F, Ribecco AS, Putzu C, Del Mastro L, Rossi E, Daniele B, Mosconi AM, Di Rella F (2018). The HOBOE-2 multicenter randomized phase III trial in premenopausal patients with hormone-receptor positive early breast cancer comparing triptorelin plus either tamoxifen or letrozole or letrozole + zoledronic acid. Ann Oncol.

